# ‘Cardiovascular Pandemic’ in Argentina

**DOI:** 10.5334/gh.843

**Published:** 2020-07-30

**Authors:** Ignacio Martín Bluro, Daniel José Piñeiro, José Luis Navarro Estada

**Affiliations:** 1 Specialization Course in Cardiology, Instituto Universitario del Hospital Italiano de Buenos Aires, AR; 2 Argentine Society of Cardiology, AR

**Keywords:** Pandemic, COVID-19, Coronavirus, Cardiovascular Health, Public Health

Throughout the 20th century, there has been a large transformation of the public health landscape leading to an increase in longevity worldwide, of which Argentina was no exception [1,2]. In fact, median life expectancy in Argentina in the year 1900 was just 41 years, with infectious diseases being the main cause of death. Vaccine development, the use of antibiotics and improvements to the healthcare system during the last Century led to an epidemiologic transition that allowed for an important improvement in life expectancy (80 years for females and 73.2 years for men in 2019) and positioned the cardiovascular disease (CVD) as the main cause of death throughout the nation [3]. The appearance of the coronavirus disease (COVID)-19 pandemic represents a sudden change in this trend and a new challenge for the healthcare system. After 60 years of a landscape dominated by non-communicable diseases such as CVD, a new infectious disease appeared worldwide and threatens even the most solid healthcare systems. There is a two-pronged hazard of this pandemic: The first hazard is the direct damage caused by the virus itself. As of May 23rd, 2020, SARS-CoV-2 infected over 5.1 million people and caused over 330,000 deaths worldwide [4]. The second hazard is the psychological effect on the population due to the physical distancing measures required to contain the virus propagation, which had a severe negative impact on health management behaviors that, as a result of multiple educational campaigns, were grounded in our Society [5]. Recent publications from both Europe and the USA already show a 40% reduction in the number of primary angioplasties due to acute coronary syndrome with ST segment deviation [6,7,8]. Similarly, a recent communication from China shows that, during the COVID-19 pandemic, patients suffering from heart attacks waited four times longer than the historic average time to call for medical help [9]. One probable explanation for these findings is that many people, because of fear for the virus, either will not consult a physician or will delay the consultation. This appears to be an imminent threat from a cardiovascular perspective.

## What Is the Status of the Pandemic in Argentina?

In 2016, the improvement of the universal healthcare coverage was established as one of the strategic policies by the Argentine government. This policy was intended to ensure that every person can receive all healthcare services that may be required, with adequate access and quality of care, and without incurring in financial hardships to obtain it [10]. However, this objective is far from being reached, and today the Argentine healthcare system is very fragmented: 60% of the population uses employer-based coverage, 30% accesses the public health system, and 10% obtained direct private coverage [11]. This fragmentation represents a barrier to the implementation of mechanisms for universal access to all the healthcare subsystems. For example, in these times in which telemedicine is becoming an alternative to safely connect patients with physicians, few providers have the required infrastructure to provide this service [12]. The few existing telemedicine platforms belong to private healthcare systems, which prevents access to those vulnerable and with limited financial resources. Moreover, because the vast majority of healthcare providers do not yet recognize telemedicine as a medical service, those physicians providing it may not be remunerated for their work. This lack of access not only deprives the patient from access to a physician, but also limits access to medications and treatment continuity.

A recent report by ADECRA+CEDIM, an industry group that represents private healthcare systems nationwide, revealed a 62% decline in consultations to emergency rooms due to coronary syndrome and a 46% decrease in calls for stroke, which led to a 59% reduction in the number of coronary angioplasties and a 58% reduction in central cardiac surgeries [13]. In a national survey conducted by the Institute of Cardiovascular Medicine of the Italian Hospital of Buenos Aires, of 6176 responses, 38% declared a personal weight increase, and 10% admitted to an increase in alcohol consumption during this pandemic. Only 57% of people with hypertension controlled their blood pressure at least once, and of those 17% declared a higher blood pressure compared to before the pandemic. Even more alarming is the fact that half the people that required medical attention during this time did not consult a physician, and most of them did not do it due to fear or lack of access to the healthcare system. Twenty-two percent of those that could not access healthcare considered that this interruption would have negative consequences to their health. Moreover, 20% had difficulties obtaining prescriptions for their medications, and 5% abandoned at least one medication. These results were even more worrisome among the most vulnerable: unemployed people, those with a lower educational level and those without health insurance, highlighting one of the common characteristics of all South American countries: inequality (Table 1) (Bluro IM et al., unpublished data).

**Table 1:** Population behaviors & perceptions according to vulnerability*.

	Total (%) (n = 6176)	No Vulnerable (%) (n = 5313)	Vulnerable (%) (n = 863)	p

Increased salt intake^1^	180 (10.9)	143 (10,2)	37 (14,8)	0.03
Fear to consult	2150 (34.8)	1779 (33.4)	371 (43.0)	<0.001
Difficulty getting prescription^2^	610 (19.4)	506 (18.4)	104 (25.5)	0.001
Medication discontinuation^3^	149 (4.7)	115 (4.2)	34 (8.3)	<0.001
Desire or need to consult	2085 (33.8)	1752 (33.0)	333 (38.6)	0.002
Avoided consulting^4^	1000 (48.1)	809 (46.3)	191 (57.4)	<0.001
Negative health consequences^5^	281 (21.8)	218 (20.6)	63 (27.0)	0.032

* Vulnerability is defined as being unemployed, not having finished high school, or not having health insurance. ^1^ Increased salt intake among hypertensive; ^2^ Difficulty obtaining a prescription among those who are medicated; ^3^ Medication discontinuation between medicated; ^4^ Avoided consulting among those who desired or need to consult; ^5^ Among those who had difficulties accessing the health system. Bluro IM et al., Unpublished data.

In times in which cardiologists are used to seeing crowded offices, emergency rooms, and coronary units, a new question arises: Where are the patients? [14] Given that the main cause of death in Argentina is CVD, this is not a trivial question. Of the 336,823 deaths occurred in the country in 2018, 95,826 were due to cardiovascular causes [15]. According to the IMPACT-CHD ARGENTINA study, there was a 30% reduction in cardiovascular mortality between 1995 and 2010. Approximately half of that reduction could be attributed to therapeutic procedures such as angioplasties and surgeries, while a third was due to a better control of risk factors such as arterial hypertension, dyslipidemias, and diabetes [16]. These data imply that, if controls of cardiovascular health remain unattended due to COVID-19 concerns, we can expect an increase of several thousand deaths from cardiovascular causes.

With 7,805 confirmed cases, 366 deaths and 2569 recovered patients [17], as a result of the safety measures imposed nationally, the Argentine healthcare system is far from collapsed. To the contrary, at the moment only half of the ICU capacity is in use [18]. In this scenario, is it now the time to procrastinate the treatment to cardiovascular patients? Shouldn’t we think instead that there is a category of cardiovascular procedures that are ‘non-deferrable elective’ (e.g., symptomatic aortic stenosis, abdominal aorta aneurisms over 55 mm, etc.) that would be advisable to approach early, even if COVID-19 cases increase? We tend to adopt the European situation as our benchmark, but the epidemiological reality today in Argentina is very different and as such it may provide an opportunity for innovative approaches. We propose to implement a resource utilization strategy based on their availability and rate of use in a given region at any given time. The underutilization of the available capacity with the expectation of a potential future collapse due to COVID-19 carries the hazard of a future saturation not only due to COVID-19, but also due to unattended traditional pathologies. In this regard, the Argentine Society of Cardiology and the Argentine Cardiology Foundation alerted the healthcare authorities about the ominous projections of future cardiovascular mortality and morbidity. They have also lobbied for an inclusion of a variety of specialists, not just infectious disease specialists, in the crisis committees advising the authorities. Similarly, the Argentine Society of Cardiology produced a document declaring their position for the management of CVD during COVID-19, which includes concrete recommendations to ensure the continuity of the cardiovascular health management in a safe manner for healthcare providers and patients [19].

Evidently, the dynamics of care for patients with CVD have changed, and cardiologists must adapt to the new norm. At the same time, our society cannot abandon the advances in prevention and treatment of CVD. Without creating a new dilemma between COVID-19 and CVD management, the solution must be framed within a collaborative paradigm in which all aspects of individual health are being considered.
